# Long term (over five-year) outcomes of thermal ablation vs surgical resection for T1N0M0 papillary thyroid carcinoma in danger triangle: a propensity-matched dual-center study

**DOI:** 10.3389/fendo.2026.1887923

**Published:** 2026-10-19

**Authors:** Danling Zhang, Sheng Chen, Jianchuan Yang, Yuhan Qiu, Zhenlong Zhao, Lingpeng Tang, Ting Hu, Ming-an Yu, Songsong Wu

**Affiliations:** 1 Department of Ultrasonography, Shengli Clinical college of Fujian Medical University, Fuzhou University Affiliated Provincial Hospital, Fujian Medical University Union Hospital, Fuzhou, China; 2 Department of Ultrasonography, Fujian Medical University Union Hospital, Fuzhou, China; 3 Department of Ultrasonography, Shengli Clinical college of Fujian Medical University, Fuzhou University Affiliated Provincial Hospital, Fuzhou, China; 4 Department of Interventional Medicine, China-Japan Friendship Hospital, Beijing, China

**Keywords:** papillary thyroid cancer, progression-free survival, surgical resection, thermal ablation, thyroid danger triangle

## Abstract

**Objective:**

To evaluate long-term efficacy and safety of ultrasound-guided Thermal Ablation(TA) versus Surgical Resection(SR) for T1N0M0 papillary thyroid carcinoma in the thyroid danger triangle.

**Materials and methods:**

This dual-center retrospective study included patients treated with TA or SR between September 2016 and April 2020. Propensity score matching (PSM) was used to balance baseline characteristics. Outcomes included overall disease progression (Regional lymph node metastasis, local tumor progression (LTP), residual tumor, distant metastasis), disease-free survival (DFS), complication rates, hospital stay duration, procedure time, blood loss.

**Results:**

After PSM, 84 patients (age 46.06 ± 10.64; 16 males) in the SR group and 84 patients(age 44.25 ± 12.67; 14 males) in the TA group were observed for a median of 67 months (range, 60–83months) and 68 months (range, 60–105 months), respectively. No significant differences were observed in overall disease progression (6.0% vs 4.8%, P = 0.732), Regional lymph node metastasis (1.2% vs 3.6%, P = 0.306),local tumor progression (LTP) (3.6% vs 1.2%, P = 0.173), or DFS (94% vs 95.2%, P = 0.898). Compared with SR, the TA group had significantly lower complication rates (6.0% vs 16.7%, P = 0.049), shorter hospital stays (median 1 vs 3.3 days, P<0.001), reduced procedure time (23 vs 82 min, P<0.001), less blood loss (1.94 vs 19.76 mL, P<0.001).

**Conclusion:**

Thermal ablation demonstrated no statistically significant difference in long-term oncologic outcomes compared with surgery for T1N0M0 PTC in the thyroid danger triangle, while offering fewer complications and faster recovery.

## Introduction

Papillary Thyroid Carcinoma (PTC) is the most common type of thyroid malignancy, accounting for more than 80% of all thyroid cancers (1). In recent years, with the promotion of the minimally invasive concept in thyroid treatment, thermal ablation technology has become an effective supplementary treatment method for low-risk PTC due to its advantages of minimal trauma, rapid recovery, and excellent cosmetic results (2–7).

Papillary thyroid carcinomas (PTC) arising within the thyroid’s “danger triangle”—a confined region bordered by the posterior thyroid margin, lateral tracheal wall, and anterior esophageal surface—pose distinct technical challenges due to their proximity to critical structures such as the recurrent laryngeal nerve and parathyroid glands (8, 9). The confined space within the thyroid’s danger triangle complicates both thermal ablation and surgery, increasing the risk of injury to adjacent critical structures and residual Tumor, thereby elevating the risk of local tumor progression (LTP) (10).

To address these procedural challenges, preliminary studies have investigated a targeted thermal ablation strategy that incorporates peritracheal hydrodissection with low-power, short-electrode techniques. This approach has demonstrated technical feasibility and short-term safety in managing PTC located within the danger triangle, offering early evidence to support its clinical applicability (11, 12). However, due to the indolent nature of PTC, short-term outcomes may not reliably reflect its long-term therapeutic efficacy—particularly for lesions located within the anatomically complex danger triangle. In this region, occult regional lymph node metastasis (LNM) may escape detection during early follow-up imaging, a common limitation in post-treatment surveillance, potentially compromising long-term prognosis (13, 14). Moreover, after-operation fibrosis and scarring may disrupt local vascular architecture, elevating the risk of delayed complications such as regional ischemia or neural dysfunction (15, 16).

Despite these concerns, no comprehensive studies have systematically compared the long-term outcomes of thermal ablation versus surgical resection for PTCs located in the danger triangle. This dual-center retrospective cohort study aims to evaluate and compare five-year oncologic outcomes—including disease-free survival, distant metastasis, and local tumor progression (LTP)—as well as safety indicators such as complication rates. The findings are expected to address a critical gap in long-term evidence and support individualized treatment planning and radiologic risk stratification for high-risk thyroid lesions.

## Materials and methods

### Patients

The retrospective study was conducted with the approval of the Human Ethics Retrospective Committee of both participating hospitals(K2023-07-18), approval date: July 18, 2023, and the requirement for written informed consent was waived due to the retrospective design. This study has been reported in line with the STROCSS guidelines (17). Clinical data of 470 patients with papillary thyroid microcarcinoma (PTMC) in the thyroid danger triangle who underwent thermal ablation (TA) or surgical resection (SR) at two centers between September 2016 and April 2020 were reviewed. In routine practice, SR is the preferred treatment, whereas TA is offered to patients unsuitable for or declining surgery. The thyroid “danger triangle” (TDT) was defined as the posterior–medial anatomical compartment of the thyroid, bordered by the dorsal thyroid surface, the lateral tracheal wall, and the anterior esophageal wall. For reproducible ultrasound assessment, the adjacent thyroid capsule on transverse view was categorized into three segments: the medial capsule (adjacent to the trachea), the posterior medial capsule (adjacent to the tracheoesophageal groove and the recurrent laryngeal nerve), and the posterior capsule (adjacent to the retropharyngeal space). The spatial relationship between the nodule and the capsule was quantified by the shortest measurable distance: “abutting” was defined as 0 mm with an intact and continuous capsule, whereas “adjacent” was defined as >0 mm but <2 mm without evidence of extrathyroidal extension.

Inclusion criteria were: (1) a single tumor ≤2.0 cm; (2) location within the thyroid danger triangle; (3) pathologically confirmed PTC (fine-needle aspiration, core biopsy, or surgical specimen); or core-needle biopsy(CNB); (3) no evidence of extrathyroidal extension (ETE, no signs of unsmooth thyroid capsule or involvement of the perithyroidal tissue) on US; (4) no evidence of cervical lymph node metastasis (LNM) on US or neck CT; (5) no distant metastasis on chest CT; (6) no prior neck irradiation; and (7) ≥60 months of follow-up, including patients who experienced early disease progression and continued scheduled follow-up after receiving standard clinical management. Exclusion criteria were: (1) invasive PTMC subtype; (2) multifocal tumors; and (3) incomplete data or loss to follow-up. After screening 194 patients, 103 treated with TA and 91 with SR were included in the final analysis (**Figure 1**). Of note, 39 patients (20.1%) in the current cohort were also included in our previously published study (11), including 21 patients (20.4%) in the TA group and 18 patients (19.8%) in the SR group. All remaining patients were newly enrolled, including the entire cohort from the second center. The prior study reported short-term (36-month) outcomes from a single center, whereas the present study evaluates long-term (≥60-month) oncologic outcomes using a dual-center, propensity score–matched design. All analyses presented here were independently performed.

**Figure 1:**
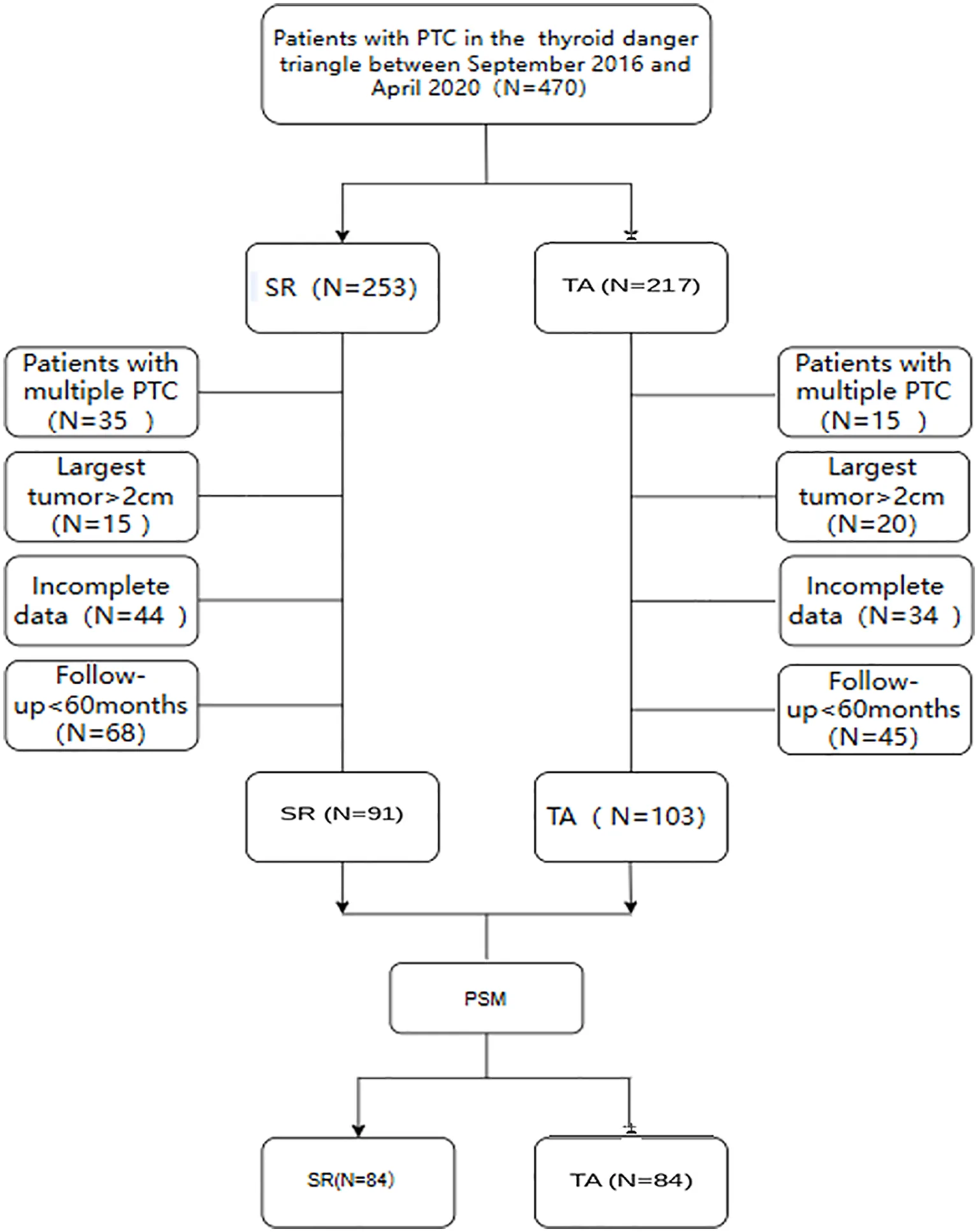
Study flowchart.

### Pre-ablation assessment

Before treatment, all patients underwent standardized preoperative evaluation, including thyroid function, parathyroid function, complete blood count, and coagulation profiles. High-resolution ultrasound, performed by two interventional radiologists with >10 years of experience, was used to assess tumor size (three dimensions), margins, vascularity, capsule integrity, and cervical lymph nodes, excluding extrathyroidal extension (ETE) and Regional Lymph Node Metastasis (LNM). Tumor volume was calculated as V= Πabc/6, where a is the largest transverse diameter and b and c are the perpendicular diameters. Additional imaging excluded nodal and distant metastases. Pathologic confirmation was obtained by ultrasound-guided fine-needle aspiration (FNA), core needle biopsy (CNB), or surgical pathology.

### Therapeutic method

Thermal ablation was performed by senior interventional radiologists with >10 years of experience under real-time ultrasound guidance and local anesthesia (1% lidocaine). Radiofrequency ablation (RFA; 5-mm active electrode, 20 W) or microwave ablation (MWA; 3-mm active antenna, 30 W) was selected based on operator preference. A trans-isthmic approach and moving-shot technique were routinely employed (**Figures 2**, **3**). To protect vital structures within the thyroid danger triangle (e.g., recurrent laryngeal nerve, trachea, esophagus), continuous hydrodissection with 5% dextrose was established to maintain a ≥5mm safety margin. Ablation margins were extended ≥3mm beyond tumor boundaries, except along the capsule. Technical success (complete avascularity) was evaluated via immediate post-procedural contrast-enhanced ultrasound (CEUS; SonoVue^®^), with supplementary ablation applied if persistent enhancement was detected. CEUS was conducted using SonoVue^®^ (sulfur hexafluoride microbubbles; Bracco Imaging, Milan, Italy) as the contrast agent. Patients were monitored for 12 hours post-procedure for potential complications.

**Figure 2:**
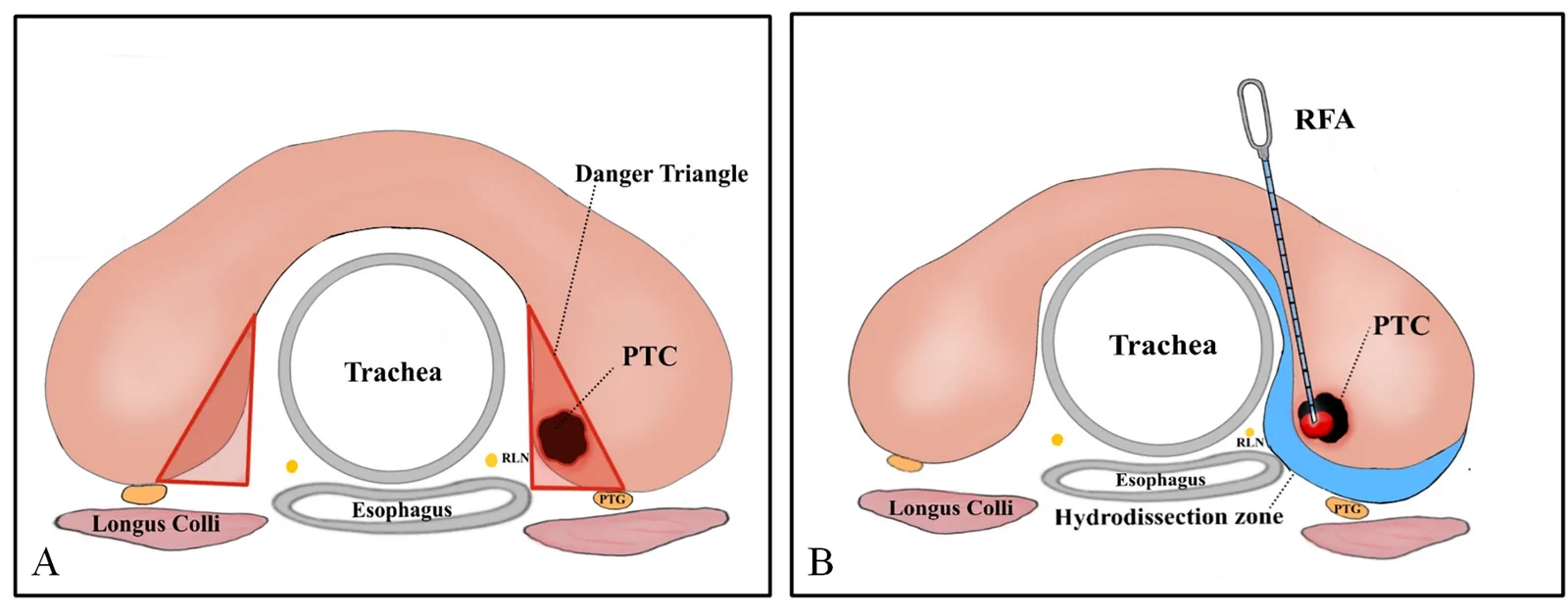
Schematic diagram of PTC in thyroid danger triangle and ablation strategy. **(A)** Schematic diagram of PTC in left thyroid danger triangle. **(B)** Schematic diagram of targeted ablation strategy combining paratracheal hydrodissection in the left side thyroid danger triangle with low-power short-electrode technique. PTC, papillary thyroid carcinoma; RLN, recurrent laryngeal nerve; PTG, parathyroid gland; RFA, radiofrequency ablation.

**Figure 3:**
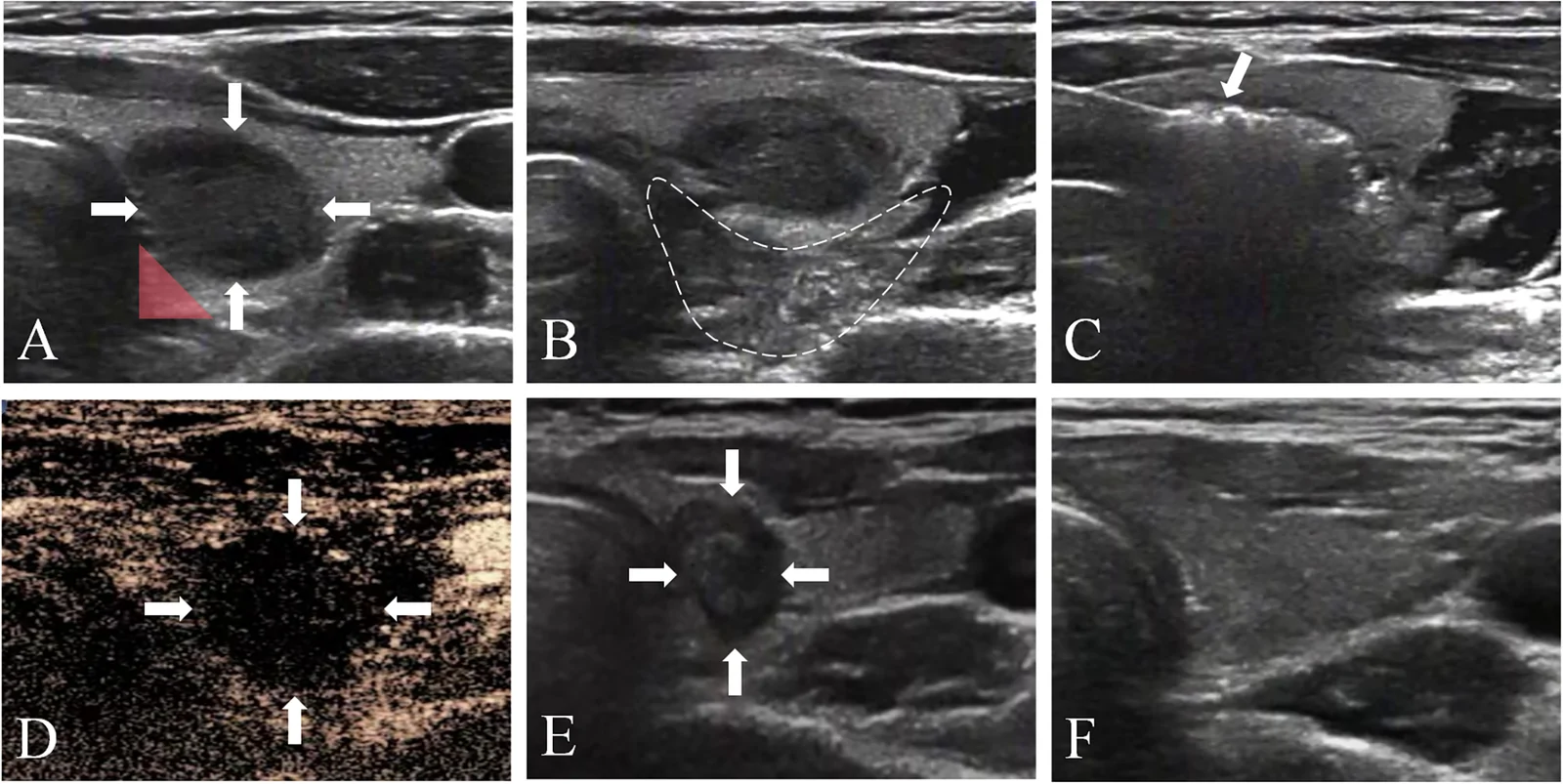
A 36-year-old woman with papillary thyroid cancer in the ‘danger triangle’ of the thyroid was treated with radiofrequency ablation (RFA). **(A)** Preoperative positioning of the target tumor (arrow), and attention is paid to avoid damaging the recurrent laryngeal nerve (red triangle) during the ablation process. **(B)** Isolation liquid injected into the posterior medial thyroid space (dotted line) to separate the tumor from adjacent structures. **(C)** Ablation continued until the tumor was fully covered by the hyperechoic zone. **(D)** Postoperative CEUS showed no enhancement, indicating complete necrosis. **(E)** Three months postoperatively, the target nodule had significantly decreased in size. **(F)** Twenty-four months postoperatively, the target nodule had completely disappeared.

Surgical resection was performed under general anesthesia by thyroid surgeons with >15 years of experience, following Chinese national consensus guidelines (18). In our cohort, all tumors were located within the thyroid danger triangle, adjacent to the posterior capsule, where the risk of central lymph node metastasis is considered higher. Therefore, in our institution, prophylactic central neck dissection (pCND) was performed as a precautionary measure. This approach was adopted to reduce the likelihood of occult nodal disease and to enhance oncologic safety in this high-risk anatomical region.

### Follow-up and evaluation

Patients in the surgical group were followed every 6–12 months, whereas the ablation group was assessed at 1, 3, 6, and 12 months, and then every 6–12 months. Follow-up included laboratory tests, ultrasound (US), and, for ablation patients, contrast-enhanced US (CEUS); annual chest CT was performed to exclude distant metastases. Metastatic lymph nodes were defined on ultrasound by the presence of one or more suspicious features, including round shape (short-to-long axis ratio <2), loss of fatty hilum, cortical thickening, microcalcifications, cystic change, or abnormal peripheral/mixed vascularity. These criteria were applied consistently during follow-up assessments to ensure accurate detection of nodal disease. Imaging evaluations focused on ablation zone size, volume, vascularity, and evidence of residual tumor, local tumor progression (LTP) or Regional Lymph Node Metastasis (LNM). Volume reduction ratio (VRR) was calculated as VRR = [(Initial Volume − Final Volume) × 100]/Initial Volume. If residual enhancement or persistent perfusion was observed on Doppler or CEUS, ultrasound-guided core needle biopsy (CNB) was performed at the ablation zone and adjacent thyroid tissue. Suspicious nodules (TI-RADS 4–5) or lymph nodes with malignant features (microcalcifications, cystic change, hypervascularity, hypo echogenicity) underwent fine-needle aspiration (FNA) or CNB. Additional imaging (CT, PET, or bone scan) was obtained when clinically indicated for suspected distant metastasis.

### Study endpoints

The primary endpoints were local tumor progression (LTP), distant metastases and disease-free survival (DFS). Local Tumor Progression was defined as (1) new thyroid malignancy confirmed by biopsy, (2) residual tumor persistent disease within the ablation zone with residual papillary thyroid carcinoma (PTC) cells on biopsy, (3) biopsy-proven local tumor progression (LTP) at the ablation margin. Distant metastasis was defined as malignant lesions outside the thyroid and regional lymph nodes, confirmed by imaging or biopsy. DFS was defined as the interval from treatment initiation to disease progression or last follow-up. Management decisions were made jointly by physicians and patients.

Secondary endpoints included treatment-related complications, procedural metrics, tumor volume changes, and parathyroid hormone (PTH) levels. Complications encompassed recurrent laryngeal nerve injury, hematoma, edema, and dyspnea. Nerve injury was defined as unilateral or bilateral vocal cord mobility restriction on laryngoscopy; persistence >6 months was considered permanent. Hypoparathyroidism was defined as PTH <15 pg/mL within 24 hours postoperatively; persistence >6 months was considered permanent. Procedural metrics included operation time (skin incision to wound closure for surgery (excluding anesthesia time); electrode insertion to completion of ablation for RFA), estimated blood loss, and hospital stay.

### Statistical analysis

The SPSS statistical software(Version 25.0) and R software (version 4.3.2) were used for statistical analyses. Continuous variables were expressed as mean ± standard deviation or median, as appropriate, and categorical variables were presented as frequencies and percentages. Comparisons of continuous variables were conducted using the Wilcoxon rank-sum test or Student’s t-test, while categorical variables were compared using the χ² test or Fisher’s exact test.

Propensity score matching (PSM) was applied to ensure baseline comparability between the surgical resection (SR) and radiofrequency ablation (RFA) groups. A 1:1 nearest-neighbor matching algorithm was used for PSM. The caliper for PSM was 0.2. Survival analysis and visualization were conducted using R software. We estimated DFS rates using the Kaplan-Meier method and compared survival distributions between groups using the log-rank test. For visualization, Kaplan-Meier curves were generated using the ‘ggplot2’ and ‘survminer’ packages, with 95% confidence intervals (CIs) displayed as shaded areas. A two-sided p-value < 0.05 was considered statistically significant.

## Results

### Patient demographics and tumor characteristics

A total of 470 patients with papillary thyroid carcinoma (PTC) located in the thyroid danger triangle were retrospectively reviewed, of whom 194 met the inclusion criteria. Among these, 103 patients (53.1%, 103/194) underwent thermal ablation (41 with microwave ablation and 62 with radiofrequency ablation), and 91 patients (46.9%, 91/194) underwent surgical resection. Propensity-score matching was performed using the following covariates: demographic (age, sex), tumor-related (location, largest diameter, initial volume, T1b classification), thyroid function (FT3, FT4, TSH, TGAb), thyroid background status, and anatomical variables (distances to the posterior capsule, anterior capsule, and tracheoesophageal groove). Among these variables, FT3, initial volume, and distance to the tracheoesophageal groove showed significant differences between the two groups before matching. After propensity score matching, 168 patients were included in the final analysis, with 84 patients in each group. Baseline characteristics were well balanced between the two groups (**Table 1**).

**Table 1:** Baseline characteristics of patients before and after propensity score matching.

Characteristic	Unmatched cohort		X^2^/t/z	P	Matched cohort		X^2^/t/z	P
	SR (n=91)	TA (n=103)			SR (n=84)	TA (n=84)		
Age((year)	46.26 ± 10.52	44.25 ± 12.67	1.193	0.234	46.06 ± 10.64	44.21 ± 12.97	1.008	0.315
Sex (%)			2.27	0.132			0.162	0.687
Male	23(25.3)	17(16.5)			16(19.0)	14(16.7)		
Female	68(74.7)	86(83.5)			68(81.0)	70(83.3)		
Location (%)			1.274	0.529			1.684	0.431
Left lobe	45(49.5)	50(48.5)			45(53.6)	41(48.8)		
Right Lobe	46(50.5)	53(51.4)			39(46.4)	43(51.2)		
FT3	4.56(4.13,4.96)	4.39(4.08,4.77)	-1.964	0.048	4.48(4.12,4.96)	4.42(4.09,4.78)	-1.123	0.261
FT4	16.96 ± 2.36	16.51 ± 2.53	1.255	0.211	16.96 ± 2.35	16.58 ± 2.68	0.967	0.335
TSH	1.86(1.42,2.71)	1.74(1.34,2.25)	-1.238	0.216	1.81(1.44,2.62)	1.77(1.38,2.3)	-0.371	0.711
TGAb	17.82(15.26,28.69)	18.21(16.08,21)	-0.310	0.757	17.81(15.42,30.22)	18.05(16.06,21.22)	-0.279	0.780
Largest diameter	0.82(0.63,1.03)	0.7(0.53,0.94)	-1.624	0.104	0.78(0.62,1.05)	0.7(0.57,0.94)	-1.391	0.164
Initial volume	0.18(0.08,0.38)	0.11(0.06,0.22)	-2.346	0.019	0.16(0.08,0.38)	0.11(0.06,0.22)	-1.916	0.055
T1b	26(28.5)	18(17.4)	3.392	0.066	25(29.7)	18(21.4)	1.532	0.213
Thyroid background status			0.586	0.444			0.249	0.618
Nomal	80(87.9)	94(91.3)			74(88.1)	76(90.5)		
Hashimoto thyroiditis	11(12.1)	9(8.7)			10(11.9)	8(9.5)		
Distance to the posterior capsule	1.8(0,3.8)	1.7(0,2.9)	-0.751	0.453	1.4(0,3.6)	1.75(0,2.9)	-0.112	0.911
Distance to the anterior capsule	2(0,4)	2.2(1,4.2)	-1.723	0.085	2.25(0,4)	2.15(0.95,4.15)	-0.948	0.343
Distance to the tracheoesophageal groove	1.5(0,2.8)	2.4(0,3.6)	-2.696	0.007	1.6(0,3)	2.4(0.5,3.55)	-1.948	0.051

p<0.05 is statistically significant.

### Intraoperative and postoperative outcomes

All 84 patients with papillary thyroid carcinoma (PTC) in the thermal ablation (TA) group underwent successful ablation in a single session. Post-procedural contrast-enhanced ultrasound demonstrated complete absence of enhancement in all target lesions, indicating a 100% technical success rate. The 84 PTC patients had an SR in a single operation in the SR, with 74 patients (88.1%) undergoing lobectomy and 10 patients (11.9%) undergoing total thyroidectomy. During a mean follow-up of 68.5 ± 8.9 months (range, 60–105 months), complete disappearance of the ablation zone was observed in 94.1% (79/84) of nodules. The disappearance rate was 96.9% (64/66) for T1a PTC and 83.3% (15/18) for T1b PTC. Median disappearance time was shorter for T1a tumors than for T1b tumors (24 vs. 36 months). Kaplan–Meier analysis demonstrated a significant difference in disappearance rates between the two subgroups (log-rank test = 14.744, p < 0.001).

In the surgical resection (SR) group, all 84 patients also completed treatment in a single operation, including 74 (88.1%) lobectomies and 10 (11.9%) total thyroidectomies, achieving a 100% technical success rate. Compared with SR, radiofrequency ablation (RFA) was associated with significantly shorter operative time, reduced postoperative hospital stay, and less blood loss. The mean incision length in the SR group was 7.24 ± 2.38 cm, whereas RFA required only a 1–2 mm skin puncture, resulting in markedly less invasiveness (**Table 2**).

**Table 2:** Comparison of treatment variables between TA and SR groups.

Parameter	T1N0M0(n=168)		P-value	T1a(n= 125)		P-value	T1b(n= 43)		P-value
	TA (n=84)	SR (n=84)		TA (n=66 )	SR (n= 59)		TA (n= 18)	SR (n= 25)	
Procedure time(min)	23.57 ± 7.31	82.32 ± 28.48	<0.001	23.41 ± 7.65	78.75 ± 18.34	<0.001	26.67 ± 7.07	90.76 ± 43.45	<0.001
Hospitalization time (d)	1 ± 0.02	3.30 ± 1.18	<0.001	1 ± 0.01	3.2 ± 1.16	<0.001	1.0 ± 0.01	3.52 ± 1.22	<0.001
Estimated Blood loss(ml)	1.94 ± 1.19	19.76 ± 10.18	<0.001	1.94 ± 1.11	19.49 ± 10.03	<0.001	1.94 ± 1.51	20.40 ± 10.69	<0.001
Incision length(cm)	0.11 ± 0.03	7.24 ± 2.38	<0.001	0.11 ± 0.03	6.71 ± 2.39	<0.001	0.11 ± 0.04	8.48 ± 1.85	<0.001

p<0.05 is statistically significant.

### Primary outcomes

Comparisons of primary outcomes are summarized in **Table 3**. In the matched cohort, no significant differences were observed between the ablation and surgical resection groups in terms of Overall disease progression (6.0% vs 4.8%, hazard ratio HR 1.26, 95% CI: 0.34–4.67, P = 0.732), Regional Lymph Node Metastasis (LNM) (1.2% vs 3.6%, HR 0.33, 95% CI: 0.03–3.21, P = 0.306), local tumor progression (LTP) (3.6% vs 1.2%, HR 3.03, 95% CI: 0.31–29.12, P = 0.173),residual tumor((1.2% vs 0%, P = 0.316)or disease-free survival (DFS) (94.1% vs 95.2%, HR 1.25, 95% CI: 0.34–4.62, P = 0.898). Disease-free survival curves rates stratified by T1, T1a, and T1b stage are shown in **Figures 4**–**6**.

**Table 3:** The comparison of disease progression between the TA and SR groups.

Event	T1N0M0 (n=168)		P-value	T1a (n= 125)		P-value	T1b (n= 43)		P-value
	TA (n=84)	SR (n=84)		TR (n=66)	SR (n= 59)		TR (n=18)	SR (n= 25)	
Total incidence	5 (6)	4 (4.8)	0.732	4 (6.1)	2 (3.4)	0.486	1 (5.6)	2 (8)	0.756
LNM	1 (1.2)	3 (3.6)	0.306	1 (1.5)	1 (1.7)	0.936	0 (0)	2 (8)	0.219
Recurrent tumors	3 (3.6)	1 (1.2)	0.173	2 (4.5)	1 (1.7)	0.366	1 (5.6)	0 (0)	0.233
Persistent tumors	1 (1.2)	0	0.316	1 (1.5)	0 (0)	0.342	0 (0)	0 (0)	NA
Distant metastasis	0 (0)	0 (0)	NA	0 (0)	0 (0)	NA	0 (0)	0 (0)	NA

p<0.05 is statistically significant.

**Figure 4:**
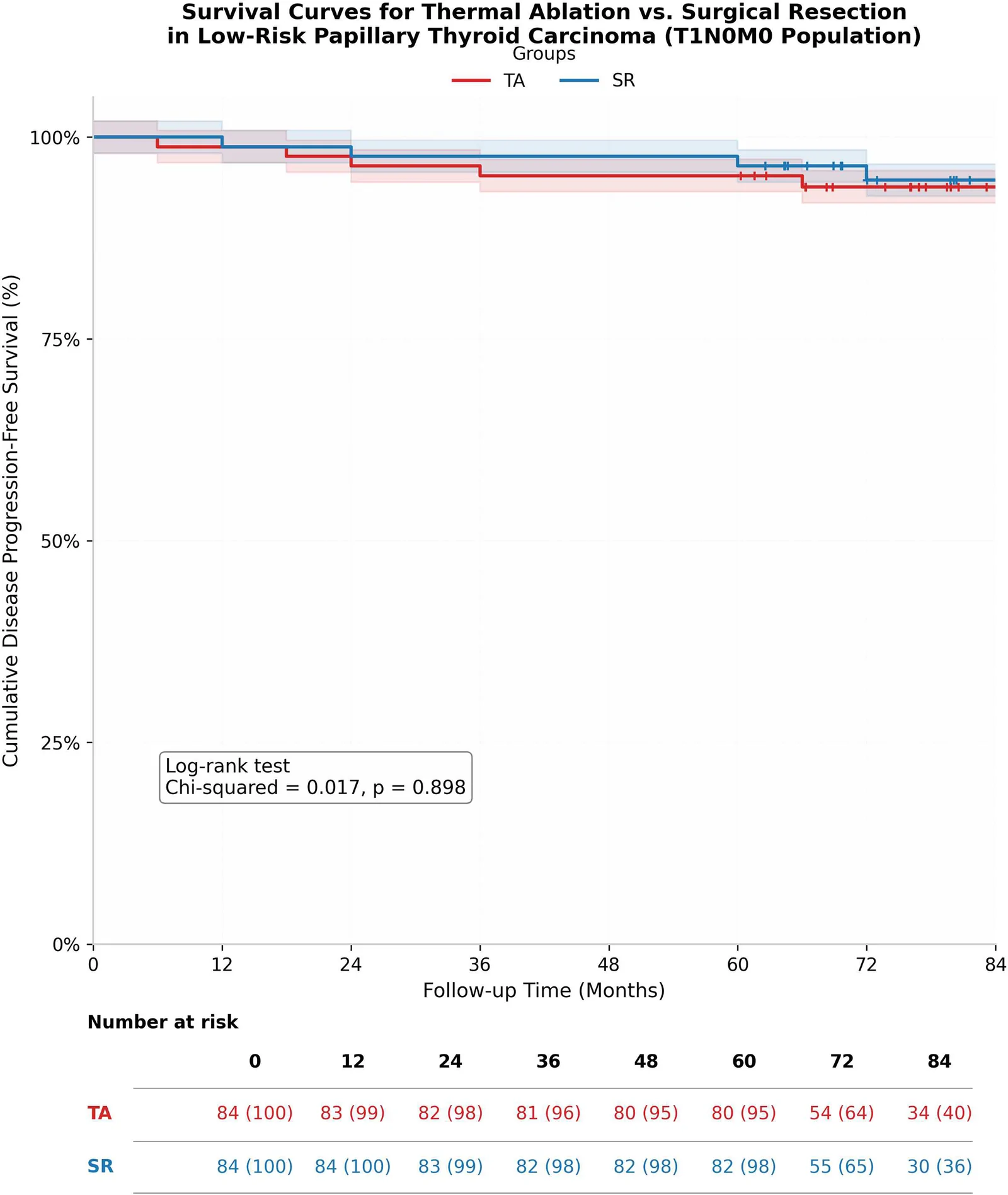
Disease-free survival curves for thermal ablation vs. surgical resection. in the treatment of low-risk papillary thyroid carcinom (T1N0M0).

**Figure 5:**
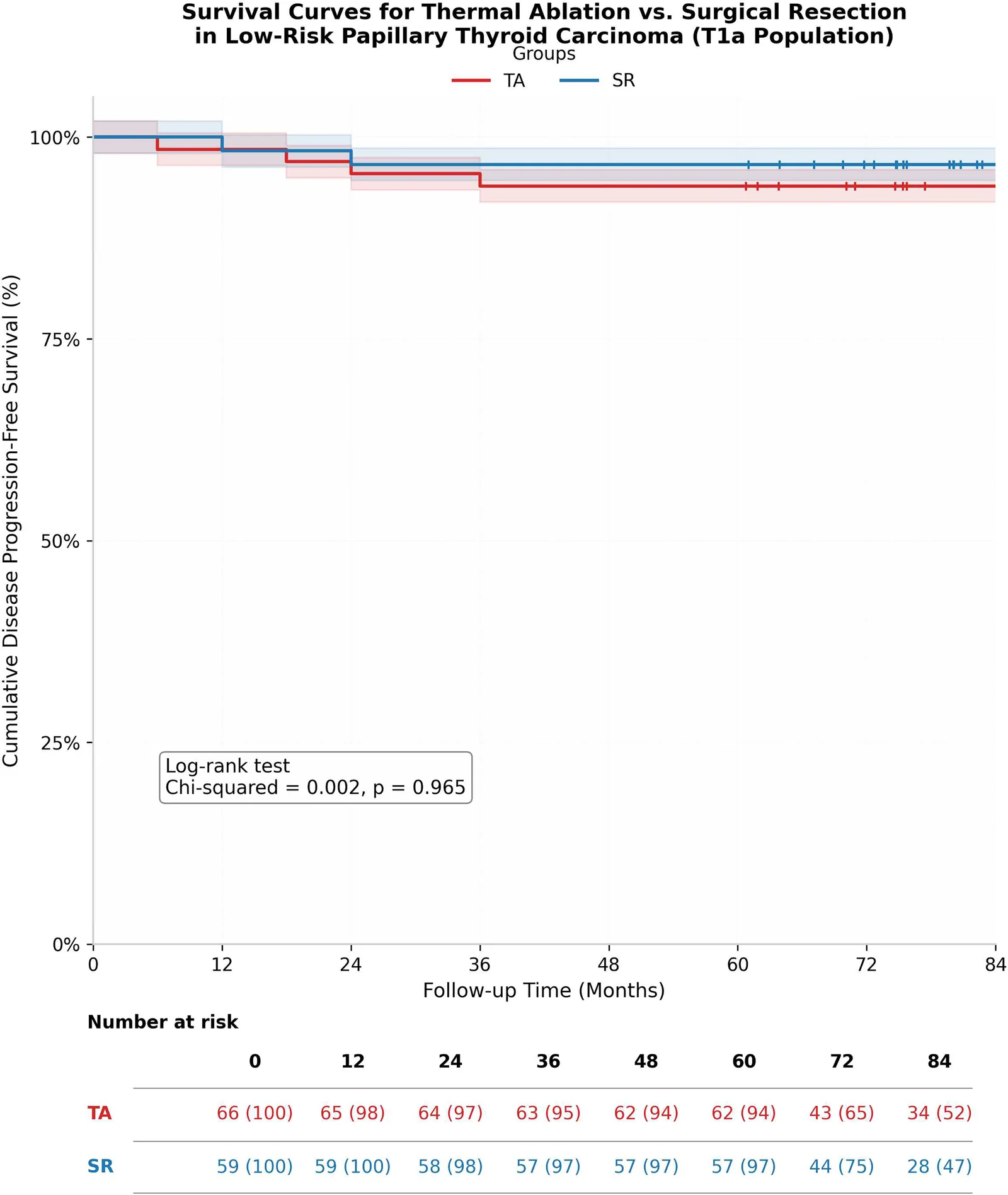
Disease-free survival curves for thermal ablation vs. surgical resection in the treatment of low-risk papillary thyroid carcinom (T1a).

**Figure 6:**
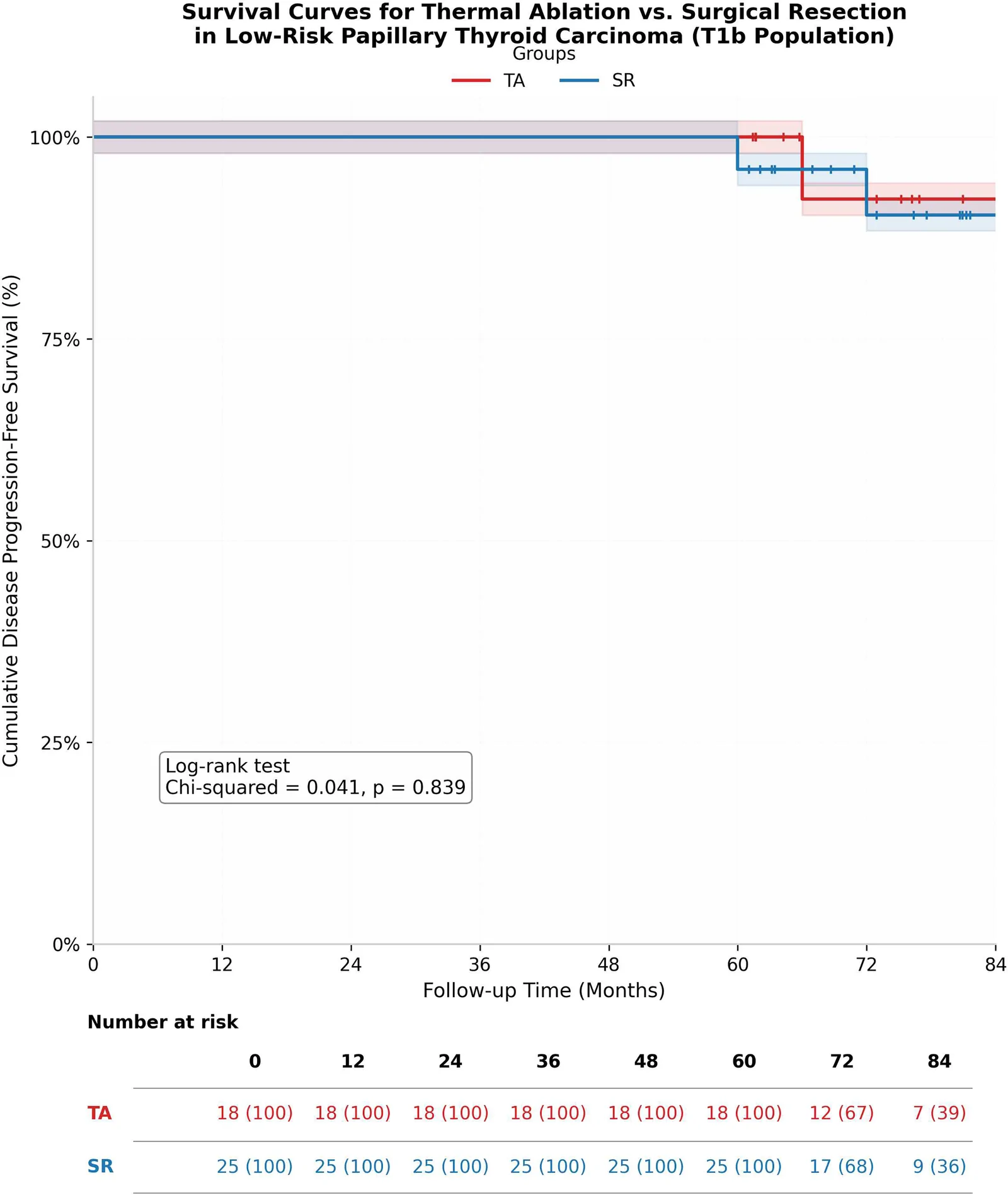
Disease-free survival curves for thermal ablation vs. surgical resection in the treatment of low-risk papillary thyroid carcinom (T1b).

In the ablation group, during a mean follow-up of 68.5 ± 8.9 months (range, 60–105 months), five patients experienced overall disease progression. Among T1a tumors, one patient developed lateral cervical lymph node metastasis, two experienced Local Tumor Progression (LTP) and one residual tumor. In T1b tumors, one local tumor progression (LTP) was observed. The single nodal metastasis involved an ipsilateral lateral cervical lymph node and was managed with additional ablation. Of the four recurrences and persistent tumor, one occurred in the contralateral lobe and three in the ipsilateral lobe; two patients underwent total thyroidectomy, one underwent additional ablation, and one opted for active surveillance.

In the surgical group, four patients developed overall disease progression. Among T1a tumors, one patient had lymph node metastasis and one had local tumor progression (LTP). Among T1b tumors, two patients developed regional LNM. Of the three nodal metastases, two involved ipsilateral lateral cervical nodes and one involved a central node. The patient with central nodal metastasis chose active surveillance. Two patients with lateral nodal metastases, who had previously undergone lobectomy only, underwent completion total thyroidectomy with unilateral lateral neck dissection.

One contralateral lobe recurrence was treated with total thyroidectomy and neck dissection, during which central nodal metastasis was also identified.

### Complications

In terms of complications, the overall complication rate was significantly lower in the TA(thermal ablation) group than in the SR (surgical resection) group (6.0% [5/84] vs. 16.7% [14/84], p = 0.049). All major complications occurred in the SR group, including 2 cases of permanent hypoparathyroidism and 4 cases of permanent hoarseness. The rate of major complications was significantly lower in the TA group than in the SR group (0% [0/84] vs. 7.1% [6/84], p = 0.028).

Regarding minor complications, there was no significant difference between the two groups (TA: 6.0% [5/84], SR: 9.5% [8/84], p = 0.564). Although the incidence of transient hoarseness was slightly higher in theTA group than in the SR group (6.0% vs. 2.6%, p = 0.441), all patients in the TA group recovered completely within 6 months without specific treatment. In contrast, 4 patients (4.8%) in the SR group experienced permanent hoarseness, although this did not reach statistical significance compared with the TA group (p = 0.120). Additionally, 5 patients who underwent total thyroidectomy in the SR group experienced transient hypoparathyroidism, a complication not observed in the TA group, resulting in a significantly higher rate in the SR group (p = 0.049). Notably, in the T1a subgroup, the overall complication rate became statistically significant after exact testing (TA: 3.0% [2/66] vs. SR: 15.3% [9/59], Fisher p = 0.026), indicating a clearer safety advantage of TA in smaller tumors. Specific comparisons of complications between the two groups are shown in **Table 4**.

**Table 4:** Occurrence of complications in the TA and SR groups.

Complication	T1N0M0 (n=168)		P-value	T1a ( n= 125 )		P-value	T1b ( n= 43 )		P-value
	TA (n=84)	SR (n=84)		TA (n= 66)	SR (n=59)		TA (n= 18)	SR (n= 25)	
Total complications	5 (6.0)	14 (16.7)	0.049	2 (3)	9 (15.3)	0.026	3 (16.7)	5 (20)	1.000
Major complications	0	6 (7.1)	0.028	0	5 (8.5)	0.022	0	1 (4)	1.000
Permanent hoarseness	0	4 (4.8)	0.120	0	3 (5.1)	0.103	0	1 (4)	1.000
Permanent hypoparathyroidism	0	2 (2.4)	0.497	0	2 (3.4)	0.221	0	0	NA
Minor Complications Hematoma	5 (6.0)	8 (9.5)	0.564	2 (3.0)	4 (6.8)	0.414	3 (16.7)	4 (16)	1.000
Transient Hypoparathyroidism	0	5 (6.0)	0.049	0	2 (3.4)	0.220	0	3 (12)	0.252
Transient Hoarseness	5 (6.0)	2 (2.6)	0.441	2 (3.0)	1 (1.7)	1.0	3 (16.7)	1 (4)	0.293
Hematoma	0	1 (1.2)	1	0	1 (1.7)	0.472	0	0	0

p<0.05 is statistically significant.

## Discussion

The thyroid danger triangle, adjacent to the recurrent laryngeal nerve, trachea, and esophagus, is widely regarded as a technically challenging region for minimally invasive therapy. In this dual-center retrospective study with propensity score matching, we compared long-term outcomes of ultrasound-guided thermal ablation (TA) and surgical resection (SR) for T1N0M0 papillary thyroid carcinoma in this high-risk area. TA and SR achieved comparable oncologic control, including overall disease progression, regional lymph node metastasis, and disease-free survival. Notably, TA demonstrated advantages in terms of lower complication rates and procedural efficiency. These findings support TA as a safe and effective alternative in anatomically complex regions and highlight the role of real-time ultrasound guidance in achieving precise and safe interventions.

Our study demonstrates that in patients with T1N0M0 PTC located in the thyroid danger triangle, TA achieves long-term tumor control comparable to surgical resection (SR). Over more than 5 years of follow-up, no significant differences were observed in overall disease progression, regional lymph node metastasis, local tumor progression (LTP), or disease-free survival (DFS), including in T1a and T1b subgroups. Overall disease progression rates increased only slightly compared with prior 3-year data (from 4.4% to 6.0% in the TA group and from 3.3% to 4.8% in the SR group); however, the magnitude of change was minimal and did not reach statistical significance, confirming durable oncologic stability (11). These findings align with long-term outcomes of TA in other thyroid regions (19–21) and mitigate concerns that the confined anatomy of the danger triangle compromises treatment completeness. Collectively, this study provides strong evidence supporting TA as a safe, effective alternative in anatomically complex regions.

From a technical perspective, the ability of TA to achieve long-term tumor control equivalent to SR relies on a comprehensive imaging-based workflow of preoperative assessment, intraoperative protection, and postoperative validation. Preoperatively, high-resolution (7–15 MHz) linear probes delineate the tumor’s relationship to the recurrent laryngeal nerve, trachea, and capsule (measurement error ≤ 1 mm), while color Doppler identifies aberrant vessels, providing the anatomical basis for the isthmus approach (22). Intraoperatively, hydro dissection with 5% glucose establishes a ≥5 mm safety margin from critical structures, followed by low-power (15–20 W), short-electrode ablation with real-time monitoring to ensure a≥3 mm margin beyond the non-capsular tumor edge (12). Postoperatively, immediate contrast-enhanced ultrasound detects residual enhancement, enabling supplementary ablation to minimize recurrence risk. The imaging-guided procedural standards not only explain the therapeutic advantages of TA in the thyroid danger triangle but also provide the essential foundation for achieving long-term tumor control comparable to surgical resection.

The incidence of complications was significantly lower in the TA group than in the SR group, with no cases of permanent recurrent laryngeal nerve injury or hypoparathyroidism observed after TA. In contrast, SR resulted in four cases of permanent hoarseness and two cases of hypoparathyroidism, reflecting the higher risk of structural disruption inherent to open surgery in the thyroid danger triangle. However, it should be noted that the difference in permanent hoarseness between the two groups did not reach statistical significance (p = 0.120). Notably, in the T1a subgroup (tumors ≤1 cm), the total complication rate in the TA group was significantly lower than that in the SR group (3.0% vs. 15.3%, p = 0.024).Given the small number of events and limited subgroup sample size, this result should be interpreted as exploratory rather than confirmatory. This exploratory signal may reflect that TA has a clear safety advantage for smaller lesions, as T1a tumors are more amenable to complete ablation under ultrasound guidance, and hydro dissection techniques can more effectively establish a safety margin, thereby reducing the risk of thermal injury to the recurrent laryngeal nerve and parathyroid glands. It is worth emphasizing that, both cases of permanent hypoparathyroidism occurred in patients who underwent total thyroidectomy due to a family history of thyroid carcinoma. The underlying cause was Inadvertent parathyroidectomy, devascularization or anatomical variation—a risk inevitably increased by the broader surgical extent even under meticulous technique—and should be interpreted as a consequence of risk-adapted surgical scope rather than the standard hemithyroidectomy approach for unilateral danger-triangle PT. In addition to the expanded resection scope in select cases, the routine use of prophylactic central neck dissection (pCND) in our surgical cohort may further contributed to the higher complication rates observed in the SR group. In the danger triangle, where tumors directly abut the tracheoesophageal groove and recurrent laryngeal nerve, surgeons at our institutions perform pCND to reduce the risk of occult nodal metastasis and avoid technically challenging reoperations in this confined compartment. While this approach enhances oncologic thoroughness, it inevitably increases the risk of transient hypoparathyroidism and nerve traction injury. Beyond these surgical considerations, the fundamental disparity in procedural safety stems from the distinct mechanisms of intervention: SR requires fascial incision and posterior thyroid dissection, increasing the likelihood of nerve traction and damage to adjacent critical structures and vessels, whereas TA-guided by high-resolution ultrasound—avoids neural pathways and employs hydro dissection with 5% glucose to establish a≥5 mm safety margin, thereby preventing thermal spread. By acting locally on the tumor, TA further minimizes collateral tissue damage, explaining the absence of major complications. In terms of efficiency, TA was associated with shorter operative time, hospital stay, and blood loss, highlighting the “precision–efficiency”advantages of minimally invasive therapy. For elderly patients or those with comorbidities, this short, low-risk approach not only lowers perioperative complications but also reduces healthcare resource utilization, underscoring its clinical adaptability.

While this study provides important evidence on the long-term comparative outcomes of TA and SR in the thyroid danger triangle, several limitations warrant discussion. First, heterogeneity in surgical extent within the SR group represents a potential source of confounding. From a real-world perspective, this variability reflects the clinical reality that surgical decisions are influenced not only by tumor characteristics but also by patient-specific risk factors such as family history. Given the small sample size, formal stratification was not feasible; therefore, we retained the matched cohort and transparently reported surgical extent to preserve statistical integrity and key complication events. Future studies with larger cohorts and standardized surgical indications are needed to further delineate the impact of surgical scope. Second, as a retrospective analysis, residual selection bias cannot be fully excluded despite propensity score matching (PSM); Unmeasured confounders—including baseline frailty, patient preferences, cosmetic concerns, and systemic comorbidities—could still influence treatment choice and recovery. Third, utilizing both radiofrequency and microwave ablation introduces technical heterogeneity due to their distinct energy mechanics and biothermal behaviors. Although sample size constraints precluded formal subgroup comparisons, this procedural variability should be considered when evaluating TA outcomes. Fourth, all TA procedures were performed by senior physicians with extensive experience in thyroid ablation. Although this strengthens internal validity, it may limit external generalizability. Given the anatomical complexity of the thyroid danger triangle, TA in this region should preferably be performed in high-volume referral centers with dedicated expertise to ensure procedural safety and reproducibility. Fifth, patient-reported outcomes such as cosmetic satisfaction and anxiety were not assessed and should be incorporated in future studies to provide a more comprehensive evaluation., Sixth differing follow-up schedules between groups (more frequent early imaging for TA) could influence the detection rate of early events, representing a potential detection bias. Moreover, because no predefined sample size calculation was conducted, the non-significant differences observed between TA and SR should not be interpreted as evidence of equivalence or non-inferiority. This statistical limitation must be considered when interpreting the comparative oncologic outcomes. Finally, the relatively small overall sample size and limited follow-up duration underscore the need for validation through larger prospective cohorts with extended follow-up.

To our knowledge, this is the first propensity score–matched study to compare long-term outcomes of TA and SR for T1N0M0 papillary thyroid carcinoma in the thyroid danger triangle. Our findings demonstrate that, under strict indications and standardized protocols, TA achieved long-term oncologic outcomes without statistically significant differences compared with surgery, while offering advantages in safety and efficiency, particularly for patients with limited surgical tolerance or cosmetic concerns. Importantly, by completely preserving thyroid function and avoiding permanent surgical morbidity or lifelong hormone replacement, TA establishes a function-sparing paradigm that empowers patient-centered shared decision-making in high-risk thyroid cancer management. By addressing a critical evidence gap in minimally invasive versus surgical management, this study provides important guidance for treatment decisions in anatomically high-risk regions. Future multicenter randomized controlled trials are warranted to validate these results in real-world settings and to expand indications, advancing the standardized use of image-guided minimally invasive techniques in thyroid cancer care.

## Data Availability

The original contributions presented in the study are included in the article/supplementary material. Further inquiries can be directed to the corresponding authors.
